# Advancing the Field of Pain Medicine—Special Issue on Pediatric Pain Management

**DOI:** 10.3390/children8030197

**Published:** 2021-03-06

**Authors:** Keri R. Hainsworth, Kristen E. Jastrowski Mano

**Affiliations:** 1 Department of Anesthesiology, Medical College of Wisconsin, Wauwatosa, WI 53226, USA; 2 Jane B. Pettit Pain and Headache Center, Children’s Wisconsin, Wauwatosa, WI 53226, USA; 3 Department of Psychology, University of Cincinnati, Cincinnati, OH 45221, USA; kristen.jastrowskimano@uc.edu

Pediatric pain management has made great strides over the past 50 years. However, considering the profound, negative effects of acute and chronic pain on youth, we must strive to improve pain management approaches for these vulnerable populations. The effects of acute pain experiences can last a lifetime and negatively affect subsequent pain experiences and well-being. For example, research has shown that postsurgical pain is undertreated in pediatric populations, and youth experience significant pain and suffering while in the hospital and after discharge. A subset of these youth go on to develop chronic pain. Chronic pain is associated with extended periods of pain and suffering and has widespread effects on functioning across physical, social, emotional, and cognitive domains. Impaired quality of life and functional disability are common, as are negative impacts on the family.

The goal of this Special Issue, *Pediatric Pain Management*, was to highlight current understanding of factors that influence both acute and chronic pain management, as well as advances in treatment across a variety of settings. We hope that readers will agree that this goal was achieved. This collection of articles represents the work of multidisciplinary teams from across the world, all striving to improve the lives of children and adolescents, and all unified by the theme of improved pain management.

In this collection, two articles focused on acute pain management–a review of challenges in measurement of patient satisfaction by Hodapp et al. [1] and an empirical study of child pain intensity and parental attitudes toward complementary medicine by Lee et al. [2]. The majority (3 reviews and 7 empirical studies) of articles in this collection focused on chronic pain.

The reviews and studies on chronic pain cover a broad range of topics. The feature article by Windsor et al., provides a much-needed narrative review of psychotropic medications for the treatment of complex pain and headache disorders in children and adolescents [3]. Other studies cover very practical aspects of treatment and testing, including a study of the clinical utility of somatosensory testing by Kersch et al. [4], and a review on the importance of pain education, by Koechlin et al. [5]. This review includes five steps that health care providers can follow when explaining chronic pain to pediatric patients and their parents. The Special Issue also included a number of articles on understudied topics within pediatric chronic pain. For example, Jastrowski Mano et al. focused on executive functioning in youth with chronic musculoskeletal pain [6]. When compared to healthy controls, youth with chronic pain showed clinically elevated impairments in working memory, inhibition, and cognitive flexibility.

We hope that readers will gain valuable information on novel and practical interventions, as well as a better appreciation for current gaps in knowledge. Continued interest and research are critical to the ongoing advancement of the field of pain medicine.
